# Should Tumor Infiltrating Lymphocytes, Androgen Receptor, and FOXA1 Expression Predict the Clinical Outcome in Triple Negative Breast Cancer Patients?

**DOI:** 10.3390/cancers11091393

**Published:** 2019-09-18

**Authors:** Anita Mangia, Concetta Saponaro, Alessandro Vagheggini, Giuseppina Opinto, Matteo Centonze, Chiara Vicenti, Ondina Popescu, Maria Pastena, Francesco Giotta, Nicola Silvestris

**Affiliations:** 1 Functional Biomorphology Laboratory, IRCCS Istituto Tumori “Giovanni Paolo II” of Bari, 70124 Bari, Italy; titasap@hotmail.com (C.S.); giusyopinto@hotmail.it (G.O.); centonzematteo@tiscali.it (M.C.); vicentichiara@libero.it (C.V.); 2 Unit of Biostatistics and Clinical Trials, Istituto Scientifico Romagnolo per lo Studio e la Cura dei Tumori (IRST) IRCCS, 47014 Meldola (FC), Italy; alessandro.vagheggin@irst.emr.it; 3 Pathology Department, IRCCS Istituto Tumori “Giovanni Paolo II” of Bari, 70124 Bari, Italy; o.popescu@libero.it (O.P.); m.irenepastena@gmail.com (M.P.); 4 Medical Oncology Unit, IRCCS-Istituto Tumori “Giovanni Paolo II” of Bari, 70124 Bari, Italy; francescogiotta@libero.it; 5 Department of Biomedical Sciences and Human Oncology, University of Bari ‘Aldo Moro’ of Bari, 70124 Bari, Italy

**Keywords:** TILs, TNBC, AR, FOXA1, prognosis

## Abstract

Tumor-infiltrating lymphocytes (TILs) are a valuable indicator of the immune microenvironment that plays the central role in new anticancer drugs. TILs have a strong prognostic role in triple negative breast cancer (TNBC). Little is known about the interaction with the androgen receptor (AR) and forkhead box A1 (FOXA1). We analyzed the relationships between TIL levels, AR, and FOXA1 expression and their clinical significance in TNBC patients. Further, we investigated their interaction with other biomarkers like programmed cell death ligand-1 (PD-L1), breast cancer type 1 susceptibility protein (BRCA1), poly (ADP-Ribose) polymerase 1 (PARP1), and Na+/H+ exchanger regulatory factor 1 (NHERF1). The expression of the proteins was evaluated by immunohistochemistry in 124 TNBC samples. TILs were performed adhering to International TILs Working Group 2014 criteria. Cox proportional hazards models were also used to identify risk factors associated with poor prognosis. Multivariate analysis identified TILs as independent prognostic factor of disease free survival (DFS; *p* = 0.045). A Kaplan–Meyer analysis revealed that the patients with high TILs had a better DFS compared to patients with low TILs (*p* = 0.037), and the phenotypes TILs−/AR+ and TILs−/FOXA1− had a worse DFS (*p* = 0.032, *p* = 0.001 respectively). AR was associated with FOXA1 expression (*p* = 0.007), and the tumors FOXA1+ presented low levels of TILs (*p* = 0.028). A poor DFS was observed for AR+/FOXA1+ tumors compared to other TNBCs (*p* = 0.0117). Low TILs score was associated with poor patients’ survival, and TILs level in combination with AR or FOXA1 expression affected patient’s clinical outcome. In addition, AR+/FOXA1+ phenotype identified a specific subgroup of TNBC patients with poor prognosis. These data may suggest new ways of therapeutic intervention to support current treatments.

## 1. Introduction

Triple-negative breast cancer (TNBC) is characterized by a lack of the estrogen receptor (ER), progesterone receptor (PgR), and human epidermal growth factor receptor 2 (Her2) expression and/or amplification [1]. TNBC constitutes 15–20% of all breast cancers (BCs), having worse prognosis and a more aggressive clinical behavior respect to non-TNBCs [2,3,4]. Chemotherapy for TNBCs remains still the standard treatment for the lack of specific molecular targets [5]. Therefore, there is an urgent need to identify new therapeutic approaches for the management of these patients. In the landscape of possible therapies new targets are emerging next to conventional treatment.

The importance of the immunologic control on cancers has long been recognized, as well as the immune-escape resulting from different factors. These remarks are true for all cancer types as much as for TNBCs [6,7]. High tumor infiltrating lymphocytes (TILs) density has been associated to a better prognosis also in TNBCs and to an enhanced pathological complete response [8]. Now seems clear that high TILs presence makes a more favorable tumor microenvironment, affecting tumor progression also in an adverse subset of tumors such as TNBCs [9]. Observations of TILs in cancer [10,11] and their association with a favorable prognosis have changed the view about this disease [12]. Current studies give hope that a thorough knowledge of the interaction between tumor cells and the immune system might lead to clinically useful biomarkers. Among these are TILs and biomarkers related to the immune/tumor interaction, such as programmed cell death ligand-1 (PD-L1). TNBCs present more mutations that are involved in the synthesis of abnormal proteins; these can act as cancer neoantigens and trigger the antitumor immune response [13]. A recent in vitro study demonstrated the therapeutic efficacy of targeting PD-L1 with specific monoclonal antibodies (mAbs) in TNBCs, showing after the treatment a down-regulation of genes promoting cell migration, invasion and metastasis, epithelial-mesenchymal transition, cell growth, survival, and hypoxia. Conversely, an up-regulation of DNA repair genes was reported [14]. Further, Raninga and colleagues proved that a therapeutic combination treatment with anti-PD-L1 antibody reduced the tumor growth in a mouse model [15]. Therefore, TNBCs can be considered an interesting subset for the development of immunotherapy.

These data underline the pivotal role of the immune system in cancer progression and therapy response, by supporting the use of tumor immune system biomarkers in clinical practice and the immunotherapy as a promising treatment strategy for this subtype of patients.

**Figure 1:**
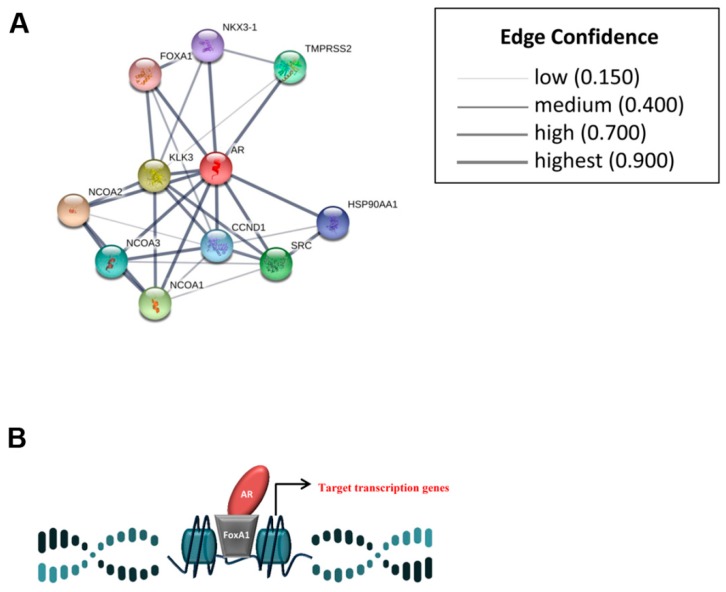
Scheme of androgen receptor (AR) transcriptional activity. (**A**) The scheme shows AR and its main interactors. A high confidence protein–protein interaction network is generated with STRING software. The network nodes are input proteins. The edges represent the predicted associations. (**B**) The interaction AR–Forkhead box A1 (FOXA1) is represented: FOXA1 should direct AR to sites usually occupied by ER, leading to an increase of proliferation estrogen-like.

In addition, to immune checkpoint markers, whose role has become increasingly important, other biomarkers have assumed considerable significance, such as the androgen receptor (AR) and forkhead box A1 (FOXA1). In the TNBC subgroup the co-expression of AR and FOXA1 has a prognostic value [16]. AR modulates the transcription of different genes by DNA-binding-dependent and independent mechanisms [17], including immune response genes [18]. Its expression is reported both in BC and in specific in TNBC [19], but the clinical significance of AR is still an open question. Recently AR has been associated to cancer cell growth and survival in TNBC cell lines and chemo-resistance in breast cancer models in vitro and in vivo [20,21,22]. Further, it is well-known that AR regulates transcriptional activity by different collaborative transcription factors, including FOXA1 [23] (Figure 1). FOXA1 has been identified as a transcriptional regulator of some liver-specific genes. It is expressed in different tumors, including BC and can bind to the promoters of more than hundred genes associated with regulation of cell signaling and the cell cycle, including ER. However, its role is unclear, some studies reported that FOXA1 and ERα constitute a major proliferative and survival axis for BC [24,25,26,27]. A recent research found that breast cancer type 1 susceptibility protein (BRCA1) was related with the suppression of FOXA1 expression in BC cell lines and that BRCA1 mutation was linked to FOXA1 promoter methylation and silencing in BCs [28]. *BRCA1* is a suppressor gene, whose dysfunction is linked to a higher risk of developing cancer, such as inhibition of DNA repair enzymes poly (ADP-Ribose) polymerase 1 (PARP1) [29]. Moreover, our team has shown in TNBC tumors that the association between nuclear PARP1 and cytoplasmic NHERF1 (Na+/H+ exchanger regulatory factor 1) expression, a scaffolding protein with oncogenic activity [30], identified a subgroup of patients with a shorter survival [31].

In this study, we explored the significance of TILs, AR, and FOXA1 expression and their impact on the clinical outcome of primary TNBC patients. Furthermore, we investigated their correlation with immunological (PD-L1), DNA repair (BRCA1, and PARP1), and progression (NHERF1) biomarkers expression.

## 2. Results

### 2.1. Protein Expression Profiling of AR, FOXA1, PD-L1, BRCA1, PARP1, and NHERF1

The expression of AR, FOXA1, PD-L1, BRCA1, PARP1, and NHERF1 was evaluated according to their specific cut-off as described in the Material and Methods section.

**Figure 2:**
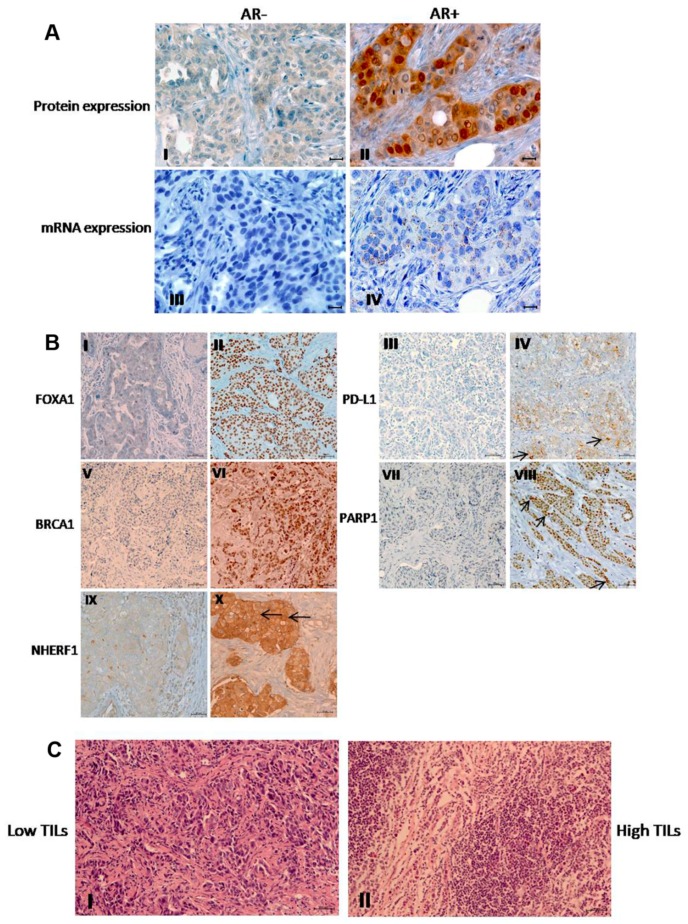
Immunohistochemical expression. (**A**) Androgen receptor (AR) I) negative and II) positive immunohistochemical protein staining and the corresponding III) negative and IV) positive mRNA xpression detected by an RNA Scope. Scale bars = 20 μm. (**B**) Representative images of immunohistochemical staining for Forkhead box A1 (FOXA1), programmed cell death ligand-1 (PD-L1), breast cancer type 1 susceptibility protein (BRCA1), poly (ADP-Ribose) polymerase 1 (PARP1), and Na+/H+ exchanger regulatory factor 1 (NHERF1) proteins. I) Negative and II) positive nuclear FOXA1 expression. III) Negative and IV) positive PD-L1 expression, the positivity has been considered for tumor cells showing partial or complete membrane staining at any intensity (Arrows). V) Negative and VI) positive high nuclear BRCA1 expression. VII) Negative and VIII) positive nuclear PARP1 expression (Arrows). IX) Negative and X) positive high membranous and cytoplasmic NHERF1 expression (Arrows). Scale bars = 50 μm. (**C**) Representative tissue samples with I) low tumor-infiltrating lymphocytes (TILs) and **II**) high TILs density. TILs were performed in full-face hematoxylin and eosin-stained sections. Scale bars = 50 μm. Images were obtained on an Axion Image 2 upright microscope (Zeiss, Oberkochen, Germany) with an Axiocam 512 color camera.

AR and FOXA1 expression was evaluated at the nuclear level in the whole cohort. Among the stained BC samples, AR was present in 87% (108/124) of tumors and the 14.8% (16/108) of these tumors were AR+. The RNAscope assay confirmed the immunohistochemistry data, showing AR mRNA expression in the same tumor samples (Figure 2A).

FOXA1 was present in 91.1% (113/124) of tumor samples and it was overexpressed in 32.7% (37/113) of them. The PD-L1 reaction remained confined to the cell membrane. In 90.3% of the tumors (112/124) PD-L1 expressing the percentage of PD-L1 positive tumor cells was 25.9% (29/112). BRCA1 expression was assessed in 106/124 (85%) of the tumors and it was positive in 60/106 (56.6%) of cases. PARP1 expression was assessed in 107/124 (86.3%) of tumor samples, and it was positive in 20/107 (18.7%) of the tumors. NHERF1 expression was detected in the apical membrane, cytoplasm, and nucleus of tumor cells. These different localizations were scored separately and their significance was evaluated. Positive membranous NHERF1 (mNHERF1) was present in 35.6% (42/118), cytoplasmic NHERF1 (cNHERF1) in 52% (61/118), and nuclear NHERF1 (nNHERF1) in 17% (20/118) of cases. 

Some examples of FOXA1, PD-L1, BRCA1, PARP1, and NHERF1 staining patterns and tissue samples with low and high TILs presence are shown in Figure 2B,C respectively. 

### 2.2. Relationship Between Tumor Markers Expression and Clinicopathological Features

A summary of significant associations between tumor marker expressions and clinicopathological features is listed in Table S1a,b. Negative FOXA1 expression showed a significant association with higher tumor histological grade (*p* = 0.016) in 88.2% of tumors. In addition, negative PD-L1 was observed in 95.2% of invasive ductal carcinomas (*p* = 0.048) and all positive PD-L1 cases were significantly associated with high proliferative activity (*p* = 0.041). High nNHERF1 expression was present in 75% of older patients (*p* = 0.004), while the lack of nNHERF1 was noticeably associated with pre-menopausal status (*p* = 0.004) in 73.5% of cases.

### 2.3. Association Between Protein Expressions Analyzed

**Figure 3:**
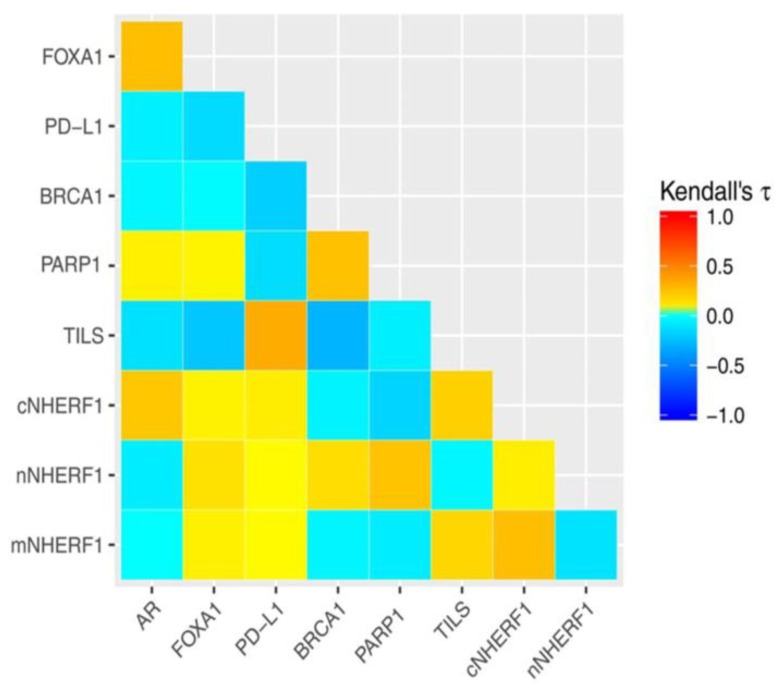
Protein interaction. Heatmap of the protein–protein interaction. AR: Androgen receptor; FOXA1: Forkhead box A1; PD-L1: Programmed cell death ligand-1; BRCA1: Breast cancer susceptibility protein 1; PARP1: Poly (ADP-Ribose) polymerase 1; TILs: tumor-infiltrating lymphocytes; mNHERF1: Membranous Na+/H+ exchanger regulatory factor 1; cNHERF1: cytoplasmic NHERF1; nNHERF1: Nuclear NHERF1.

We also analyzed the association among the tumor biomarkers. The statistical analyses, using continuous variables, showed a direct significant association between AR and FOXA1 (τ = 0.354; *p* < 0.001) and cNHERF1 expression (τ = 0.241; *p* = 0.002). BRCA1 was directly correlated to PARP1 expression (τ = 0.248; *p* = 0.001), and PARP1 was directly linked to nNHERF1 (τ = 0.297; *p* < 0.001) and inversely to mNHERF1 expression (τ = −0.242; *p* = 0.003). When we analyzed TILs correlation, there was a direct relation comparing TILS with PD-L1 and cNHERF1 expression (τ = 0.259; *p* = 0.001; τ = 0.199; *p* = 0.004 respectively) and inverse relation between TILS and BRCA1 expression (τ = −0.172; *p* = 0.020). Statistical analysis and its relative heatmap are shown in Figure 3 and Table 1. These results were also confirmed by dichotomized variables.

### 2.4. Expression of Proteins and Patient Clinical Outcome

Univariate analyses were carried out for all the clinicopathological characteristics and the expression of AR, FOXA1, PD-L1, BRCA1, PARP1, mNHERF1, cNHERF1, and nNHERF1 proteins, as dichotomized and continuous variables. These were correlated to disease free survival (DFS) and overall survival (OS). Considering dichotomized variables, no significant differences were observed in the DFS and in the OS analyses among patients with high and low AR, FOXA1, PDL1, and NHERF1 protein expression. We found a significant association between BRCA1 and PARP1 with OS (*p* = 0.030, *p* = 0.032, respectively). Moreover, the subgroup of patients with high TILs had a better 5-year DFS and OS compared to patients with low TILs, 84% vs. 75% (*p* = 0.037) and 98% vs. 88% (*p* = 0.019) respectively (Table 2).

Multivariate analysis identified the TILs as an independent prognostic factor of DFS (HR = 0.34, 95% confidence interval (CI): 0.12–0.98, *p*=0.045, (Table 3)).

**Figure 4:**
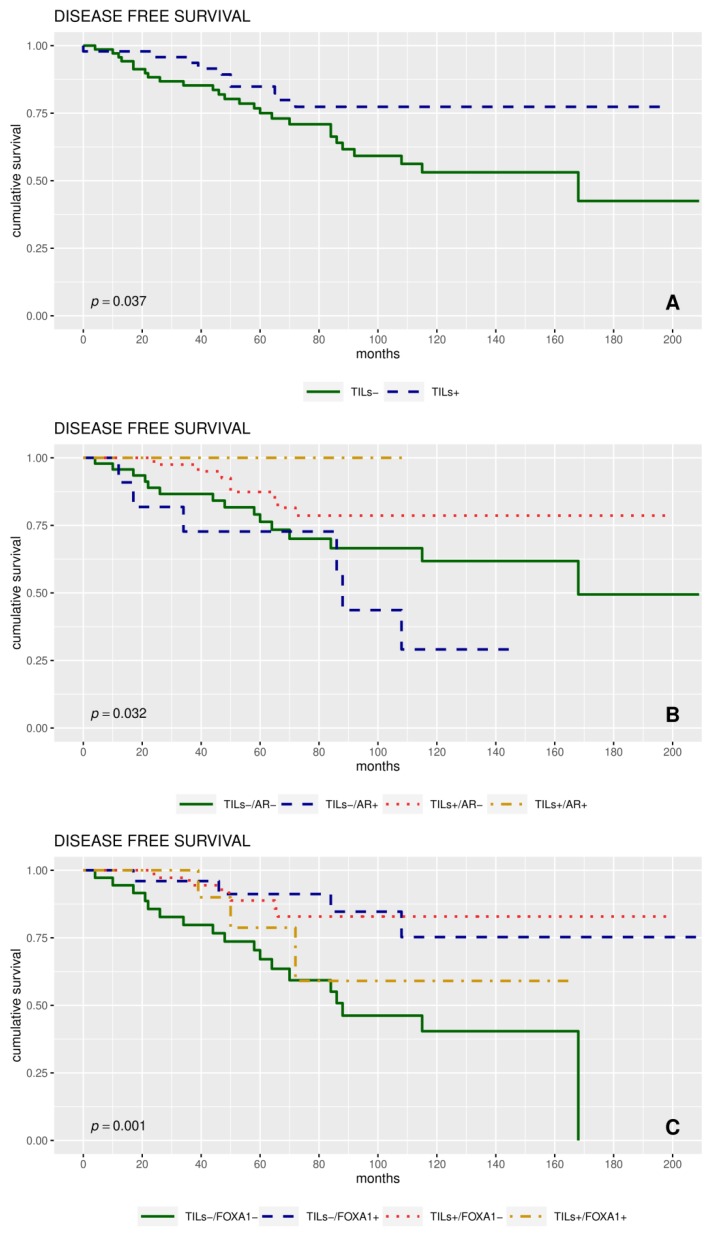
Survival analyses. (**A**) Disease-free-survival (DFS) curves for patients with tumor-infiltrating lymphocytes positive (TILs+; reverse Kaplan–Meier method median follow up: 108, 95% confidence interval (CI) (85–140); events: 10/47) versus TILs- (median follow up: 104, 95% CI (86–142); events: 26/70) presence (*p* = 0.037). (**B**) DFS curves for patients with simultaneously TILs–/androgen receptor (AR)– (median follow up: 104, 95% CI (77–151); events: 15/47), TILs–/AR+ (median follow up: 142, 95% CI (61-not available (NA); events: 6/11), TILs+/AR− (median follow up: 118, 95% CI (89–143), events: 8/40), TILs+/AR+ (median follow up: 84, 95% CI (77–NA); events: 0/5) expression: The four Kaplan–Meier curves resulted significantly different (*p* = 0.032). (**C**) DFS curves for patients with simultaneously TILs−/forkhead box A1 (FOXA1)– (median follow up: 110, 95% CI (68–164); events: 18/36), TILs−/FOXA1+ (median follow up: 92, 95% CI (62–147); events: 4/26), TILs+/FOXA1− (median follow up: 117, 95% CI (89–143); events: 6/36) and TILs+/FOXA1+ (median follow up: 110, 95% CI (60–NA); events: 3/10) expression: The four Kaplan–Meier curves resulted significantly different (*p* = 0.001).

Next, we investigated the relationship between protein expression and TNBC patient’s survival. Kaplan–Meier curves revealed that the patients with higher TILs score had a better DFS (*p* = 0.037; Figure 4A).

Considering co-expression markers:

TILs−/AR−, TILs−/AR+, TILs+/AR−, and TILs+/AR+, Kaplan–Meier analysis showed that the patients with TILs−/AR+ tumors had a worse DFS respect to the other phenotypes considered (*p* = 0.032; Figure 4B).

TILs−/FOXA1−, TILs−/FOXA1+, TILs+/FOXA1−, and TILs+/FOXA1+ phenotypes, we observed significant differences in DFS among four groups. The subgroup TILs−/FOXA1−, had a worse DFS (*p* = 0.001), compared to other groups (Figure 4C). 

Moreover, a poor DFS was observed for AR+/FOXA1+ tumors respect to other tumors (*p* = 0.0117; Figure S1). The patients with AR+ and FOXA1− tumors tended toward a poorer DFS than patients with AR−/FOXA1+ tumors (*p* = 0.080). The subgroup TILs−/BRCA1+ showed a worst DFS respect to TILs+/BRCA1− (*p* = 0.097). Moreover, no significance was found considering the TILs+/PD-L1+ phenotype (*p* = 0.100; Figure S2).

## 3. Discussion

The recent scientific research has drawn particular attention to the identification of predictive/prognostic biomarkers for targeted analysis and therapy on TNBCs. Therefore, it is necessary to better understand the molecular regulatory mechanisms of this type of BC, in order to develop new effective therapeutic approaches to improve patients’ treatment. 

The prognostic relevance and the potential predictive impact of TILs in TNBCs have been recognized. Different studies confirmed that high levels of TILs are associated with better survival [16,32,33,34].

In our series, multivariate analysis revealed that TILs were an independent prognostic factor of DFS, and, Kaplan–Meier analysis showed that TNBC patients with high TIL levels had a better DFS.

In addition, when TILs were considered in association with AR, Kaplan–Meier analysis revealed a worse DFS for the TILs−/AR+ than other phenotypes. This result reinforced the key role that AR could play in more aggressive tumors and endorsed the protective action of TILs. A protective role was demonstrated by TILs also in the subgroup phenotype TILs+/AR+, balancing the hypothetical negative role of AR and highlighting a subgroup with no recurrence. Patients with TILs−/FOXA1− tumors had a shorter prognosis than other subgroups, suggesting a pivotal role of the immune infiltrate. However a meta-analysis study also showed that low FOXA1 expression level was associated with a poor survival outcome [35].

Our results showed AR expression in about the 15% of cases, with cut-offs of 10%, unlike Guiu [16]. The choice of a better cut-off is still subject of comparison. We adopted a cut-off of 10% following careful review of the literature [36,37]. In agreement with previous studies, we observed a poor DFS in AR+ TNBCs, rationalizing a pharmacological AR block as a potential endocrine therapy for these patients [16,38]. The literature disagrees about the prognostic impact of AR in TNBC. In fact, some authors found an association of AR with a better prognosis in BC [38,39,40,41]. Heterogeneity of used antibodies, chosen cut-offs and patient cohorts make the AR expression particularly variable and its prognostic role controversial [42].

We underlined a significant association AR/FOXA1 and a shorter DFS in AR+/FOXA1+ phenotype, despite the small number of cases, in accordance with other authors [16]. Moreover, the involvement of AR in the tumor aggressiveness is also highlighted by the correlation between AR and cNHERF1, a marker of tumor progression in BCs [31,43], and involved in different signaling pathway [44,45]. Interestingly, we observed a direct interaction between TILs and cNHERF1, that could appear a non-sense being two markers with an antagonist action: Protective and tumorigenic, respectively. Nevertheless, recently we demonstrated a pivotal role of NHERF1 in the orchestrate tumor microenvironment signaling pathways, with a strictly association with angiogenic factors and epithelial-mesenchymal transition proteins [30]. Its involvement in the tumor inflammatory panorama could create the favorable environment to recall TILs migration in the major cancer areas.

It is well known that dysfunction of DNA repair systems such as BRCA1 or PARP1 are involved in carcinogenesis [29]. In this cohort of TNBCs, no significant statistically associations emerged among analyzed biomarkers. We confirmed a direct correlation between BRCA1 and PARP1 expression, to prove the up-regulation of these two repair systems, particularly in TNBCs [31]. In addition, an inverse relationship between BRCA1 and TILs was found, hypothesizing that BRCA1-mutated tumors could have more tumor-specific neoantigens and, therefore, increased TILs. This is in line with what is reported by Massink, who observed that BRCA1-mutated BCs were affected by the presence of high numbers of TILs [46]. The survival analyses were made for all markers alone and in combination with TILs, but no significant results were observed in the different subgroups: TILs-NHERF1, TILs-BRCA1, and TILs-PARP1 phenotypes.

The PD-1/PD-L1 pathway is an important immunosuppression mechanism by which cancer cells escape host immunity [47].

Some recent studies described a higher PD-L1 expression in TNBCs [16,48], but the rate of expression is extremely variable [49], and its prognostic significance is still debated. These differences might be caused by evaluation of PD-L1 expression and the cut-off values adopted, antibodies used and in the type of sample analyzed. In the present study, about 26% of TNBCs expressed PD-L1 and it was associated to tumors with high proliferative activity, but no with AR no FOXA1. The DFS of patients with TILs+/PD-L1+tumors was improved, as reported by other authors [50], even if our result was not statistically significant.

We are aware that changes of standard treatments of this tumor along the time of collection of tumor specimens could represent a potential limit of our study. On the other side, this retrospective series allowed us to have available a very long follow up for these patients.

## 4. Materials and Methods

### 4.1. Patients and Clinicopathological Characteristics 

A total of 124 primary TNBC patients who had received surgical treatment at the IRCCS, Istituto Tumori “Giovanni Paolo II” of Bari, Italy between 1996 to 2012, were retrieved from a data base of the Pathology Department of our institute. The patients were selected retrospectively according to the availability of the biological material and the clinical follow up. Our patient series was not consecutive. Characteristics of patients are shown in Table 4. All patients received adjuvant chemotherapy (anthracycline and taxane). The patients were selected according to the following criteria: (i) Female gender; (ii) histologically confirmed invasive carcinoma with estrogen (ER) and progesterone (PgR) receptor-negative tumors (<1% of positive cells), and any HER2 status (absence of HER2 overexpression or amplification), (iii) no evidence of distant metastasis at diagnosis, (iv) no treatment of any type prior to surgery, and (v) no patients with a history of previous malignancies. ER, PgR, proliferative activity, and HER2 status were provided by the Pathology Department of our institute. ER and PgR assessment used the ER/PgR (PharmDX kits, Dako, Santa Clara, CA, USA). HER2 status was evaluated using a monoclonal antibody (MoAb clone CB11; Novocastra Laboratories, Ltd., Newcastle, UK) and scored in accordance with the HercepΤest scoring system (Food and Drug Administration, Silver Spring, MD, USA) [51]. HER2 was considered to be positive if immunostaining was 3+ or if a score 2+ showed gene amplification by fluorescence in situ hybridization (FISH). Results were reported using American Society of Clinical Oncology and the College of American Pathologists (ASCO/CAP 2007 criteria) [52]. Ki67 nuclear staining was used to assess the proliferative activity, with a cut off value of 20% positive cells to indicate the tumors with Ki67 >20% as highly proliferating. The analysis of TILs was assessed in full-face hematoxylin and eosin sections, according to the International TILs Working Group 2014 criteria [53]. Tumors with TILs score of ≥50% were considered lymphocyte predominant breast cancer. The study was approved by the Ethics Committee of the Istituto Tumori “Giovanni Paolo II” with the reference 657/CE on 13th December 2018.

### 4.2. TMA and Immunohistochemistry

Tissue microarrays (TMAs) were assembled from formalin-fixed and paraffin-embedded (FFPE) tissues of tumors using the Galileo Tissue MicroArrayer CK 4500 (Transgenomic, Hillington Park, Glasgow, UK). Each sample was arrayed in triplicate to minimize tissue loss and to overcome tumor heterogeneity.

Four-micrometer-thick sections were cut from FFPE blocks and mounted onto slides. The slides were processed and stained as previously reported [31].

The OptiView DAB IHC Detection Kit (Ventana Medical Systems, Tucson, AZ, USA) was used to detect protein expression and for PD-L1 staining an OptiView Amplification Kit (Ventana Medical Systems, Tucson, AZ, USA) was also used. All solutions were from Ventana Medical Systems unless otherwise specified. Briefly, slides underwent deparaffinization with the EZ PREP solution, followed by antigen retrieval with Cell Conditioning solution 1 at 95° for BRCA1 (60 min), AR (56 min), FoxA1 (32 min), and PD-L1 (32 min), and Cell Conditioning solution 2 at 95 °C for PARP1 (36 min). No antigen retrieval was executed for NHERF1. The following step was incubation at 37° with the following specific primary antibody diluted in Antibody Diluent: Rabbit polyclonal NHERF1 antibody (anti-EBP50; ThermoFisher Scientific, Rockford, IL, USA), 1:350 (16 min); mouse monoclonal PARP1 antibody (F-2 clone, Santa Cruz Biotechnology Inc., Santa Cruz, CA, USA), 1:500 (16 min); mouse monoclonal BRCA1 antibody (MS110 clone; Oncogene Research Products, Calbiochem, Merck KGaA, Darmstadt, Germany), 1:75 (32 min); mouse monoclonal anti-human PD-L1 (clone 22c3, Dako Agilent, Santa Clara, CA, USA), 1:100 (32 min); mouse monoclonal anti-FoxA1 (clone 2f83, Merck Millipore, Burlington, MA, USA), 1:200 (36 min); and mouse monoclonal anti-human androgen receptor (clone AR441, Dako agilent, Santa Clara, CA, USA), 1:50 (36 min). Finally, tissues were counterstained with hematoxylin and a bluing reagent for 8 min and 4 min respectively, then were dehydrated and mounted. Positive and negative controls were included in each staining run as indicated in the data sheet of each antibody. All antibodies used in this study have been validated in the pre-analytic phase to guarantee a satisfactory level of reproducibility and accuracy.

### 4.3. Immunohistochemical Assessment

To analyze protein expression of AR, FoxA1, PD-L1, BRCA1, PARP1, and NHERF1 we utilized TMAs including 124 breast cancer samples. For immunohistochemical assessment we followed the previous method [31]. The data from the immunohistochemistry assay were examined independently by two of the researchers. If one core was uninformative, lost, or contained no tumor tissue, the overall score applied was that of the remaining cores. For each analyzed protein, the cases in which all three cores were uninformative were considered non-assessable and excluded from the analyses. Any discrepancies between the two observers were resolved by re-examination and consensus. The examination of AR expression was assessed on the basis of nuclear staining intensity and we used 10% as cut-off value (negative <10; and positive ≥10) [37].

For nuclear FoxA1 staining, the percentage of positive cells was estimated and the average intensity was scored similarly to PARP1. In this quickscore (QS) system a final score was calculated by multiplying the percentage score by the intensity score. The percentage of staining was categorized as: 0 = no nuclear expression; 1 = 1% to 10% positive tumor nuclei; 2 = 11% to 20%; and so on until a maximum score of 10 = 91% to 100% positive tumor nuclei. The intensity was scored as: 1+ = weak staining; 2+ = moderate staining; and 3+ = strong staining. A QS between 0 and 3 was classified as negative, and a score ≥4 was considered positive [54]. PD-L1 protein expression was determined by using the tumor proportion score (TPS), which is the percentage of viable tumor cells showing partial or complete membrane staining at any intensity. A TPS ≥1% was referred to as positive staining for PD-L1 [55]. The immunohistochemical assessment of BRCA1, PARP1, and NHERF1 was scored as previously reported [31,56] and it was detailed in Table S2.

### 4.4. RNA Scope

For evaluation of AR mRNA expression a commercial kit (RNAscope® 2.5 High Definition (HD)-BROWN Assay, Advanced Cell Diagnostics, Newark, CA, USA) was used. Paraffin embedded samples were cut into 5 +/− 1 μm sections, baked for 1 h at 60 °C and deparaffinized. Subsequently the slides were pretreated with a RNAscope® Pretreatment Kit (Advanced Cell Diagnostics, Newark, CA, USA) target retrieval solution and Protease Plus) to unmask target RNA and permeabilize cells. The probe was then hybridized to target mRNA for 2 h at 40 °C. The signal was amplified using a multi-step process, followed by hybridization to horseradish peroxidase (HRP)-labeled probes. The signal was detected using a chromogenic substrate, 3,3′-diaminobenzidine (DAB) for 10’ at room temperature (RT), and before mounting the slides were counterstained with 50% hematoxylin I for 2’ at RT and washed in a 0.02% ammonia bath for 10 seconds. Tissue sections were examined under a standard bright field microscope at 20–40× magnification. Positive signals were visible as brown punctate dots.

### 4.5. Follow Up and Statistical Analysis 

The Chi-squared test and Kendall rank test were assessed to evaluate the tumor markers relationship using categorical variables and continuous variables, respectively. Protein expression analyses were carried out in relation to disease-free survival (DFS) and overall survival (OS) in months. DFS was described as the timeframe between diagnosis and loco-regional/distant relapse (second invasive BC, second primary cancer, and/or death without evidence of BC to the date of last contact). OS was described as the timeframe between diagnosis and date of last contact or of death from any cause. Univariate analyses of biomarkers expression and clinicopathological variables were performed for both DFS and OS, using the Kaplan–Meier method (5-year, 95% confidence interval (CI)) and compared by the log rank test. The hazard ratio (HR) for univariate and multivariate analyses was calculated by the Cox proportional hazard regression model. Statistical significance level was *p*-values < 0.05. Statistical analyses were achieved using the statistical packages survival and survminer of the statistical language R, version 3.4 (R Core Team: Vienna, Austria) [57].

## 5. Conclusions

Our data proved that low TILs score was associated with poor patients’ survival, and the presence or absence of TILs in combination with AR or FOXA1 expression affected the patient’s clinical outcome. Moreover, AR+/FOXA1+ phenotype identified a specific subgroup of TNBC patients with a poor DFS. The improved DFS of the TILs+/PD-L1+ tumor phenotypes (even though not statistically significant) suggests a general activation of immune system in TNBCs, highlighted also by direct correlation between TILs and PD-L1. Our results suggest that TILs may be a good marker of the immune response, and underline the need of future studies on the relationship between the immune system and cancer cells.

## Figures and Tables

**Table 1:** Non-linear relationship between two continuous variables assessed by the Kendall rank test.

	AR		FOXA1		PD-L1		BRCA1		PARP1		TILS		cNHERF1		nNHERF1	
	τ	*p*-value	τ	*p*-value	τ	*p*-value	τ	*p*-value	τ	*p*-value	τ	*p*-value	τ	*p*-value	τ	*p*-value
**FOXA1**	0.354	<0.001														
**PD-L1**	−0.022	0.803	−0.110	0.173												
**BRCA1**	−0.068	0.425	−0.038	0.623	−0.164	0.054										
**PARP1**	0.126	0.143	0.080	0.307	−0.046	0.595	0.248	0.001								
**TILS**	−0.116	0.145	−0.124	0.085	0.259	0.001	−0.172	0.020	−0.003	0.966						
**cNHERF1**	0.241	0.002	0.129	0.073	0.138	0.079	−0.060	0.418	−0.051	0.496	0.199	0.004				
**nNHERF1**	−0.071	0.429	0.098	0.229	0.064	0.462	0.134	0.106	0.297	<0.001	−0.040	0.602	−0.057	0.455		
**mNHERF1**	0.037	0.668	0.011	0.888	−0.029	0.732	−0.004	0.959	−0.242	0.003	0.168	0.024	0.221	0.002	−0.111	0.177

AR: Androgen receptor; FOXA1: Forkhead box A1; PD-L1: Programmed cell death ligand-1; BRCA1: Breast cancer susceptibility protein 1; PARP1: Poly (ADP-Ribose) polymerase 1; TILs: Tumor-infiltrating lymphocytes; mNHERF1: Membranous Na+/H+ exchanger regulatory factor 1; cNHERF1: Cytoplasmic NHERF1; nNHERF1: Nuclear NHERF1.

**Table 2:** Univariate analysis on categorical data.

Characteristics	pts.	DFS				OS		
		Events	5-year DFS (95% CI) ^a^	HR (95% CI) ^b^	*p*-Value ^c^	Events	5-year OS (95% CI) ^a^	*p*-Value ^c^
**Overall**	124	36	80 (73–88)			12	92 (87–97)	
Age								
≤51	65	22	79 (70–90)	1.00	0.432	5	93 (87–100)	0.460
>51	59	14	81 (71−92)	0.765 (0.39–1.50)		7	89 (82-98)	
Pre/post								
Post	73	25	81 (73–90)	1.00	0.810	7	94 (88–99)	0.391
Pre	41	11	79 (66–93)	1.09 (0.53–2.23)		5	87 (77–98)	
Size								
≤2 cm	59	15	87 (78–96)	1.00	0.552	4	93 (86–100)	0.431
>2 cm	64	21	74 (64–86)	1.22 (0.63–2.38)		8	90 (83–98)	
Histological type								
IDC	111	33	81 (74–89)	-	0.947	9	93 (89–98)	**0.027**
ILC	4	1	75 (43–100)	-		0	100 (100–100)	
Other	9	2	74 (48–100)	-		3	67 (42–100)	
Lymph node status								
Negative	65	16	83 (74–93)	1.00	0.578	6	90 (83-98)	0.894
Positive	55	17	79 (69–91)	1.21 (0.61−2.40)		6	92 (85–100)	
Ki67								
Negative (≤20%)	12	4	82 (62–100)	1.00	0.843	1	100 (100–100)	0.945
Positive (>20%)	110	30	81 (73–89)	0.90 (0.32–2.56)		11	90 (85–96)	
AR								
Negative (<10%)	92	23	83 (75–91)	1.00	0.219	7	93 (88–99)	0.111
Positive (≥10%)	16	6	81 (64–100)	1.75 (0.70–4.33)		3	87 (71−100)	
FOXA1								
Negative	76	24	79 (71−89)	1.00	0.281	8	90 (84–97)	0.990
Positive	37	7	88 (77–100)	0.63 (0.27–1.47)		4	91 (82–100)	
PD-L1								
Negative (<1%)	83	24	83 (75–92)	1.00	0.460	7	94 (88–99)	0.432
Positive (≥1%)	22	7	79 (66–96)	0.73 (0.31−1.69)		4	86 (75–100)	
BRCA1								
Negative (<3%)	46	11	84 (74–96)	1.00	0.273	1	100 (100–100)	**0.030**
Positive (≥3%)	60	18	82 (72–93)	1.52 (0.72–3.23)		8	88 (80–97)	
PARP1								
Negative (0–9)	87	24	83 (75–91)	1.00	0.523	6	95 (91−100)	**0.032**
Positive (10–18)	20	6	85 (71−100)	1.34 (0.55–3.28)		4	80 (63–100)	
TILs								
Negative (<50%)	70	26	75 (65–87)	1.00	0.037	10	88 (80–96)	**0.019**
Positive (≥50%)	47	10	84 (75–96)	0.47 (0.23–0.971)		1	98 (94–100)	
mNHERF1								
Negative (0%)	76	19	81 (73–91)	1.00	0.478	8	89 (82–96)	0.435
Positive (>0%)	42	14	78 (66–92)	1.28 (0.64–2.57)		3	95 (88–100)	
cNHERF1								
Negative (70%)	57	14	81 (71−93)	1.00	0.645	7	87 (78–96)	0.202
Positive (≥70%)	61	19	79 (69–90)	1.18 (0.59–2.35)		4	95 (89–100)	
nNHERF1								
Negative (0%)	98	17	78 (70–87)	1.00	0.276	8	93 (87–98)	0.321
Positive (>0%)	20	3	89 (76–100)	0.52 (0.16–1.71)		3	83 (68–100)	

^a^ confidence interval (CI), five-year disease-free survival (DFS) or overall survival (OS) based on the Kaplan–Meier method, ^b^ hazard-ratio (HR) computed using the Cox proportional hazard regression model for the DFS (for OS cannot be computed due to the low number of events), ^c^ *p*-value of the log-rank test for the equality of probability of an event (relapse for DFS or death for OS). ); IDC: Invasive ductal carcinoma; ILC: Invasive lobular carcinoma; AR: Androgen receptor; FOXA1: Forkhead box A1; PD-L1: Programmed cell death ligand-1; BRCA1: Breast cancer susceptibility protein 1; PARP1: Poly (ADP-Ribose) polymerase 1; TILs: tumor-infiltrating lymphocytes; mNHERF1: Membranous Na+/H+ exchanger regulatory factor 1; cNHERF1: Cytoplasmic NHERF1; nNHERF1: Nuclear NHERF1.

**Table 3:** DFS multivariate analysis on categorical and continuous variables.

Characteristics	Categorical		Continuous	
	HR (95% CI) ^a^	*p*-Value ^a^	HR (95% CI)	*p*-Value ^a^
AR	1.65 (0.54–5.02)	0.382	1.00 (0.99–1.03)	0.509
FOXA1	0.38 (0.12–1.20)	0.100	0.64 (0.16–2.48)	0.517
PD-L1	0.49 (0.13–1.76)	0.272	1.00 (0.96–1.04)	0.863
BRCA1	1.47 (0.53–4.11)	0.459	1.00 (0.99–1.02)	0.386
PARP1	1.40 (0.46–4.32)	0.554	1.00 (0.99–1.02)	0.583
TILS	0.34 (0.12–0.98)	0.045	0.19 (0.02–1.51)	0.116
mNHERF1	2.28 (0.89–5.82)	0.085	1.01 (1.00–1.02)	0.104
cNHERF1	0.92 (0.37–2.32)	0.867	1.00 (0.99–1.02)	0.805
nNHERF1	0.62 (0.13–3.00)	0.548	0.88 (0.70–1.10)	0.263

^a^ hazard-ratio (HR), confidence interval (CI) and *p*-value for five-year disease-free survival (DFS) computed using the multivariate Cox proportional hazard regression model with categorical and continuous variables. AR: Androgen receptor; FOXA1: Forkhead box A1; PD-L1: Programmed cell death ligand-1; BRCA1: Breast cancer susceptibility protein 1; PARP1: Poly (ADP-Ribose) polymerase 1;TILs: tumor-infiltrating lymphocytes; mNHERF1: Membranous Na+/H+ exchanger regulatory factor 1; cNHERF1: Cytoplasmic NHERF1; nNHERF1: Nuclear NHERF1.

**Table 4:** Clinicopathological characteristics of 124 triple negative breast cancer (TNBC) patients.

Characteristics	*n*	(%)
Patients Age: median value 51 (range 26–80)		
≤51 years	65	(52.4)
>51 years	59	(47.6)
Menopausal status		
Pre	83	(66.9)
Post	41	(33.1)
Histological type		
IDC	111	(89.5)
ILC	4	(3.2)
Other	9	(7.3)
Histological grade		
G1	1	(0.8)
G2	23	(18.7)
G3	99	(80.5)
Unknown	1	
Tumor size (cm)		
≤2 cm	59	(48.0)
>2 cm	64	(52.0)
Unknown	1	
Lymph node status		
Negative	65	(54.2)
Positive	55	(45.8)
Unknown	4	
Ki67		
Negative (≤20%)	12	(9.8)
Positive (>20%)	110	(90.2)
Unknown	2	
TILs		
Negative (<50%)	70	(59.8)
Positive (≥50%)	47	(40.2)
Unknown	7	

IDC: Invasive ductal carcinoma; ILC: Invasive lobular carcinoma; TILs: Tumor-infiltrating lymphocytes.

## Supplementary Materials

The following are available online at https://www.mdpi.com/2072-6694/11/9/1393/s1, Table S1: Relationship between tumor markers and clinicopathological features; Table S2: Dilution, source, staining of antibodies and cut off used; Figure S1: Survival analysis. DFS curves for patients with simultaneously AR+/FOXA1+ phenotype respect to all other tumors (*p*= 0.0117); Figure S2: Survival analysis. DFS curves for patients with simultaneously AR−/FOXA1+ vs. AR+/FOXA1− (*p* = 0.080). TILs−/BRCA1+ vs. to TILs+/BRCA1− (*p* = 0.097) and TILs+/PD-L1+ Vs TILs−/PD-L1− (*p* = 0.100) expression.
