# Supplementary material for: Should Tumor Infiltrating Lymphocytes, Androgen Receptor, and FOXA1 Expression Predict the Clinical Outcome in Triple Negative Breast Cancer Patients?
Source: Cancers (Basel). 2019 Sep 18;11(9):1393. doi: 10.3390/cancers11091393 (PMC6769726; doi:10.3390/cancers11091393)
Supplement: Supplementary file 1 [file cancers-11-01393-s001.pdf]

Supplementary Material

Table S1a. Relationship between tumor markers expression and clinicopathological features.

	AR					*FOXA1*					*PD-L1*					*BRCA1*				
	**Negative**		**Positive**		***p*-value**	**Negative**		**Positive**		***p*-value**	**Negative**		**Positive**		***p*-value**	**Negative**		**Positive**		***p*-value**
Characteristics	n	(%)	n	(%)		n	(%)	n	(%)		n	(%)	n	(%)		n	(%)	n	(%)	
Patient age																				
≤ 51 years	53	(57.6)	8	(50.0)	*0.571*	44	(57.9)	16	(43.2)	*0.143*	45	(54.1)	15	(51.7)	*0.817*	23	(50.0)	30	(50.0)	*1.000*
> 51 years	39	(42.4)	8	(50.0)		32	(42.1)	21	(56.8)		38	(45.8)	14	(48.3)		23	(50.0)	30	(50.0)	
Pre/Post																				
Pre	68	(73.9)	8	(50.0)	*0.053*	55	(72.4)	20	(54.1)	*0.053*	54	(65.1)	21	(72.4)	*0.469*	31	(67.4)	38	(63.3)	*0.664*
Post	24	(26.1)	8	(50.0)		21	(27.6)	17	(45.9)		29	(34.9)	8	(27.6)		15	(32.6)	22	(36.7)	
Histological type																				
IDC	84	(91.3)	15	(93.8)		69	(90.8)	32	(86.5)		79	(95.2)	25	(86.2)		42	(91.3)	55	(91.7)	
ILC	2	(2.2)	0	(0.0)	*0.836*	1	(1.3)	2	(5.4)	*0.445*	2	(2.4)	0	(0.0)	***0.048***	2	(4.3)	1	(1.7)	*0.635*
Other	6	(6.5)	1	(6.2)		6	(7.9)	3	(8.1)		2	(2.4)	4	(13.8)		2	(4.3)	4	(6.7)	
Tumor size (cm)																				
≤2 cm	41	(44.6)	7	(43.8)	*0.952*	34	(44.7)	19	(51.4)	*0.508*	42	(51.2)	10	(34.5)	*0.121*	19	(41.3)	30	(50.8)	*0.331*
>2 cm	51	(55.4)	9	(56.2)		42	(55.3)	18	(48.6)		40	(48.8)	19	(65.5)		27	(58.7)	29	(49.2)	
Lymph node status																				
Negative	48	(53.3)	7	(46.7)	*0.632*	40	(54.1)	19	(54.3)	*0.982*	42	(53.2)	15	(51.7)	*0.894*	19	(42.2)	33	(57.9)	*0.116*
Positive	42	(46.7)	8	(53.3)		34	(45.9)	16	(45.7)		37	(46.8)	14	(48.3)		26	(57.8)	24	(42.1)	
Histological grade																				
G1	0	(0.0)	0	(0.0)		0	(0.0)	1	(2.8)		0	(0.0)	0	(0.0)		0	(0.0)	1	(1.7)	
G2	13	(14.1)	5	(31.2)	—	9	(11.8)	11	(30.6)	***0.016***	18	(21.7)	3	(10.3)	—	7	(15.6)	15	(23.3)	*0.403*
G3	79	(85.9)	11	(68.8)		67	(88.2)	24	(66.7)		65	(78.3)	26	(89.7)		38	(84.4)	45	(75.0)	
Ki67																				
Negative (≤ 20%)	6	(6.7)	1	(6.2)	*0.951*	5	(6.8)	6	(16.2)	*0.116*	11	(13.4)	0	(0.0)	***0.041***	4	(8.7)	6	(10.3)	*0.777*
Positive (> 20%)	84	(93.3)	15	(93.8)		69	(93.2)	31	(83.8)		71	(86.6)	28	(100.0)		42	(91.3)	52	(89.7)	

continued

IDC: Invasive Ductal Carcinoma; ILC: Invasive Lobular Carcinoma; AR: Androgen Receptor; FOXA1: Forkhead box A1; PD-L1: progammed cell death ligand-1; BRCA1: breast cancer susceptibility protein 1; PARP1: Poly [ADP-Ribose] Polymerase 1; Tumor-infiltrating lymphocytes (TILs); mNHERF1: membranous Na+/H+ Exchanger Regulatory Factor 1; cNHERF1: cytoplasmic NHERF1; nNHERF1: nuclear NHERF1.

Table S1b. Relationship between tumor markers expression and clinicopathological features.

	PARP1					*mNHERF1*					*cNHERF1*					*nNHERF1*				
	**Negative**		**Positive**		***p*-value**	**Negative**		**Positive**		***p*-value**	**Negative**		**Positive**		***p*-value**	**Negative**		**Positive**		***p*-value**
Characteristics	n	(%)	n	(%)		n	(%)	n	(%)		n	(%)	n	(%)		n	(%)	n	(%)	
Patient age																				
≤51 years	43	(49.4)	12	(60.0)	*0.394*	40	(52.6)	24	(57.1)	*0.638*	30	(52.6)	34	(55.7)	*0.735*	59	(60.2)	5	(25.0)	***0.004***
>51 years	44	(50.6)	8	(40.0)		36	(47.4)	18	(42.9)		27	(47.4)	27	(44.3)		39	(39.8)	15	(75.0)	
Pre/Post																				
Pre	57	(65.5)	13	(65.0)	*0.965*	53	(69.7)	27	(64.3)	*0.544*	39	(68.4)	41	(67.2)	*0.888*	72	(73.5)	8	(40.0)	***0.004***
Post	30	(34.5)	7	(35.0)		23	(30.3)	15	(35.7)		18	(31.6)	20	(32.8)		26	(26.5)	12	(60.0)	
Histological type																				
IDC	81	(93.1)	17	(85.0)		67	(88.2)	39	(92.9)		52	(91.2)	53	(86.9)		89	(90.8)	17	(85.0)	
ILC	3	(3.4)	0	(0.0)	*0.097*	2	(2.6)	1	(2.4)	*0.679*	2	(3.5)	2	(3.3)	*0.646*	3	(3.1)	0	(0.0)	*0.303*
Other	3	(3.4)	3	(15.0)		7	(9.2)	2	(4.8)		3	(5.3)	6	(9.8)		6	(6.1)	3	(15.0)	
Tumor size (cm)																				
≤2 cm	40	(46.5)	10	(50.0)	*0.778*	36	(48.0)	19	(45.2)	*0.774*	31	(55.4)	24	(39.3)	*0.617*	46	(47.4)	9	(45.0)	*0.843*
>2 cm	46	(53.5)	10	(50.0)		39	(52.0)	23	(54.8)		25	(44.6)	37	(60.7)		51	(52.6)	11	(55.0)	
Lymph node status																				
Negative	39	(46.4)	12	(63.2)	*0.188*	43	(57.3)	19	47.5)	*0.314*	31	(56.4)	31	(51.7)	*0.083*	50	(52.1)	12	(63.2)	*0.376*
Positive	45	(53.6)	7	(36.8)		32	(42.7)	21	(52.5)		24	(43.6)	29	(48.3)		46	(47.9)	7	(36.8)	
Histological grade																				
G1	1	(1.2)	0	(0.0)		0	(0.0)	1	(2.4)		0	(0.0)	1	(1.7)		1	(1.0)	0	(0.0)	
G2	20	(23.3)	1	(5.0)	*0.155*	14	(18.4)	6	(14.6)	*0.701*	10	(17.5)	10	(16.7)	*0.614*	15	(15.5)	5	(25.0)	*0.539*
G3	65	75.6)	19	(95.0)		62	(81.6)	34	(82.9)		47	(82.5)	49	(81.7)		81	(83.5)	15	(75.0)	
Ki67																				
Negative (≤20%)	11	(12.9)	0	(0.0)	*0.089*	6	(7.9)	4	(10.0)	*0.701*	5	(8.8)	5	(8.5)	*0.955*	8	(8.3)	2	(10.0)	*0.809*
Positive (>20%)	74	(87.1)	20	(100.0)		70	(92.1)	36	(90.0)		52	(91.2)	54	(91.5)		88	(91.7)	184	(90.0)	

IDC: Invasive Ductal Carcinoma; ILC: Invasive Lobular Carcinoma; AR: Androgen Receptor; FOXA1: Forkhead box A1; PD-L1: progammed cell death ligand-1; BRCA1: breast cancer susceptibility protein 1; PARP1: Poly [ADP-Ribose] Polymerase 1; Tumor-infiltrating lymphocytes (TILs); mNHERF1: membranous Na+/H+ Exchanger Regulatory Factor 1; cNHERF1: cytoplasmic NHERF1; nNHERF1: nuclear NHERF1.

Table S2. Dilution, source, staining of antibodies and cut off used.

Biomarkers	Dilution	Source/clone	Staining Localization	Cut off (range)	
**NHERF1**	1:150	Affinity Bioreagents, rabbit polyclonal EBP50, PA1-090	membrane	>0% *	(0–90%)
			cytoplasmic	≥70% *	(0–100%)
			nuclear	>0% *	(0–70%)
BRCA1	1:75	Calbiochem, mouse monoclonal anti-BRCA1, MS110	nuclear	≥3% *	(0–90%)
PARP1	1:100	Santa Cruz, mouse monoclonal, F-2 nuclear	nuclear	≥10 ** (0–	(0–18)

* median value; ** quick score method. NHERF1: Na+/H+ Exchanger Regulatory Factor 1; BRCA1: breast cancer susceptibility protein 1; PARP1: Poly [ADP-Ribose] Polymerase 1.

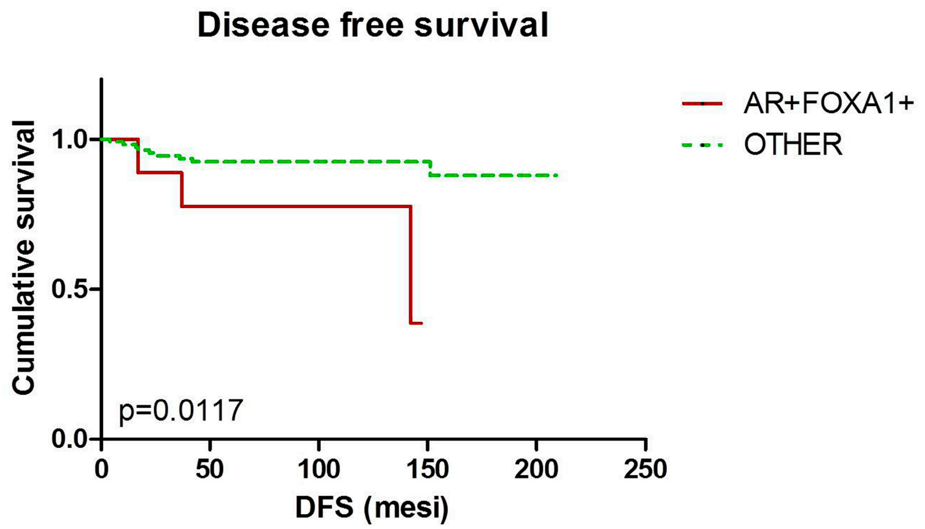

Figure S1. Survival analysis. DFS curves for patients with simultaneously AR+/FOXA1+ phenotype respect to all other tumors ($p = 0.0117$).

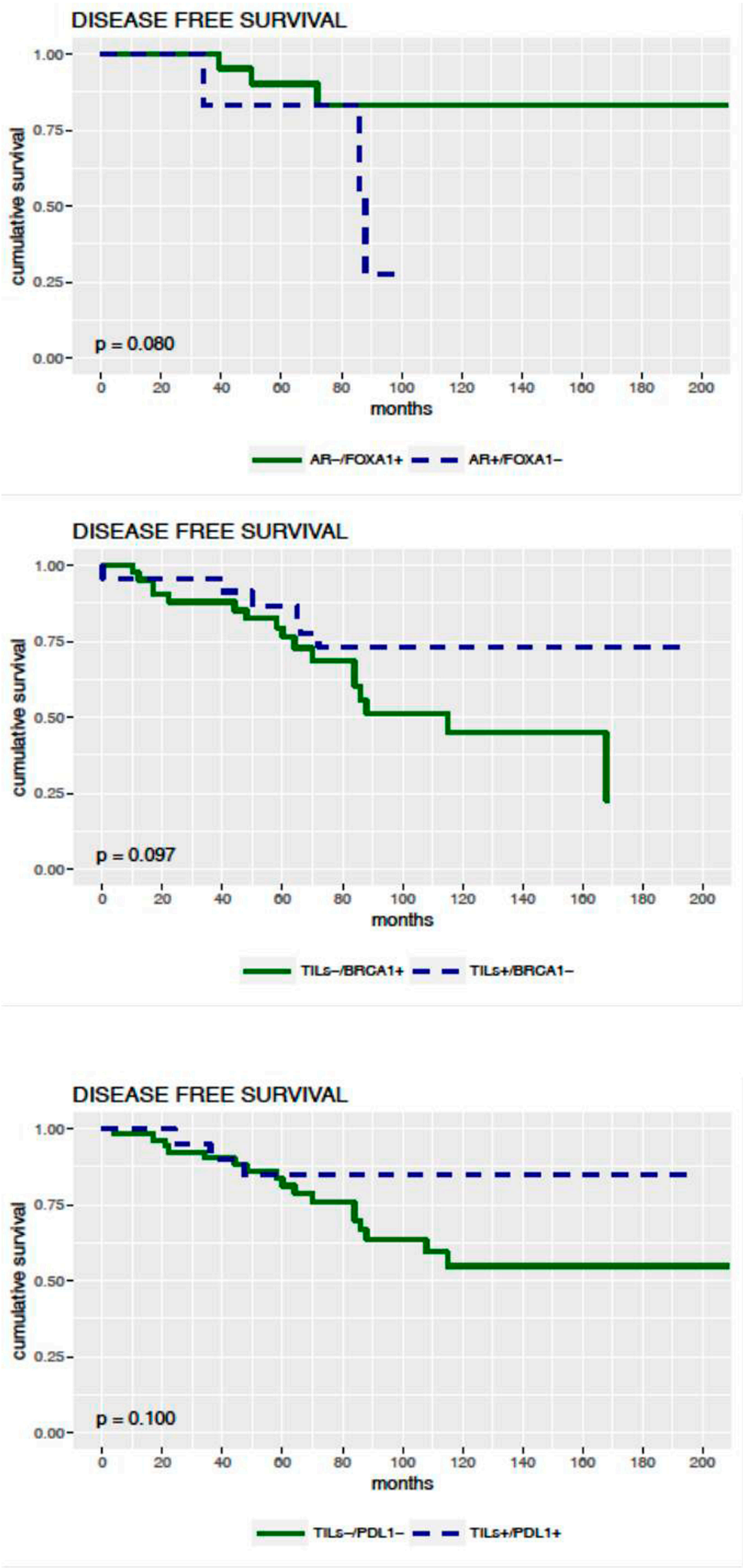

Figure S2. Survival analyses. DFS curves for patients with simultaneously AR−/FOXA1+ vs AR+/FOXA1− (*p* = 0.080). TILs−/BRCA1+ vs to TILs+/BRCA1- (*p* = 0.097) and

Table 1. Vs TILs-/PD-L1- (p = 0.100) expression.
